# Lifestyle and Social Factors Exacerbated on the Prevalence of Mood Disorders and Functional Dyspepsia Among Neonatal Nurses in China

**DOI:** 10.3389/fpsyt.2022.905401

**Published:** 2022-05-16

**Authors:** Zhen-peng Huang, Fang Huang, Mei-jun Wang, Chuan-zhuang Tang, Jiang-ping Huang, Juan Ling, Shan-e Li, Su-qiao Wei, Hai-hua Lei, Jing-jing Li, Xiu Lan

**Affiliations:** ^1^Faculty of Nursing, Guangxi University of Chinese Medicine, Nanning, China; ^2^Department of Neonatology, Nanning Maternal and Child Health Hospital, Nanning, China; ^3^Department of Community, Nanning Maternal and Child Health Hospital, Nanning, China; ^4^Department of Neonatology, Guangxi Zhuang Autonomous Region Maternal and Child Health Hospital, Nanning, China; ^5^Department of Neonatology, The Second Nanning People's Hospital, Nanning, China; ^6^Department of Neonatology, Guangxi International Zhuang Medicine Hospital, Nanning, China; ^7^Department of Neonatology, The First People's Hospital of Nanning, Nanning, China

**Keywords:** neonatal nurses, anxiety, depression, functional dyspepsia, social factors, lifestyles

## Abstract

**Background:**

Nursing is a high-stress occupation that can have an impact on mental health, particularly for neonatal nurses. Job-related stress factors and work-related behaviors have played a critical role in nurses' mental health. This study aimed to explore the prevalence of mood disorders and the impact of social factors, lifestyle on mood disorders among neonatal nurses.

**Methods:**

A total of 260 participants comprising neonatal nurses and nurses who work in neonatal intensive care units (NICU) were recruited. Data were collected using a validated generalized anxiety disorder questionnaire, patient health questionnaire-9, Pittsburgh sleep quality index, and social factors and lifestyle assessments.

**Results:**

In total, 49.23% of neonatal nurses exhibited mood disorders, particularly a combination of depression and anxiety. Female, poor interpersonal relationships and unhappy marital status, preference for smoking, alcohol, irregular diet, and poor sleep were common in neonatology nurses who exhibited mood disorders; preference for coffee and tea were lower in neonatology nurses without mood disorders (all *P* < 0.05). Interpersonal relationships, marital status, irregular diet, and poor sleep were independent factors associated with mood disorders among neonatal nurses (all *P* < 0.05). Mood disorders presented as functional dyspepsia (FD) among 50.78% of the participants (*P* < 0.05). Poor sleep and preference for smoking were common among neonatal nurses who had FD with mood disorders (all *P* < 0.05). Furthermore, the preference for sugary beverages was lower in participants with FD and mood disorders (*P* < 0.05). Poor sleep was independently associated with FD with mood disorders in neonatology nurses (*P* < 0.05).

**Conclusion:**

Prevalence of anxiety and depression was higher among neonatal nurses. Furthermore, most cases of mood disorders presented as FD. Thus, social factors and lifestyle have an impact on mood disorders which can manifest through somatic symptoms.

## Introduction

Nurses play an important role in the healthcare workforce. In addition to providing health care, nurses also assist patients during rehabilitation and impart health education to patients (1). Nursing is a high-stress occupation involving night-shift work schedules, addressing different patient needs, and irregular lifestyles that can have a severe impact on nurses' mental health (2, 3). Newborn patients are often susceptible to experiencing respiratory, nutrition, feeding, and neurologic injury problems. Furthermore, the parents also face an increased risk of perinatal mood and anxiety disorders (4, 5). Particularly, the challenges faced by nurses who work in the neonatal department and neonatal intensive care unit (NICU) are more severe and stressful, thereby significantly effecting their mental health.

Psychosomatic diseases can result from the interaction of biological, psychological, and social factors (6). Anxiety and depression are the most prevalent mood disorders associated with psychosomatic diseases (7). Psychological indicators for distress include, but are not limited to, disturbances in sleep and appetite. Furthermore, physical effects experienced by nurses may include the increased risk of functional dyspepsia (FD) (8, 9). Moreover, chronic stress may lead to or exacerbate maladaptive behaviors such as smoking, alcohol consumption, and irregular diet (10, 11). Additionally, job-related stress factors, work-related behaviors, and past experiences also play a critical role in nurses' mental health (12, 13).

Recent studies revealed a high prevalence of mood disorders, including anxiety and depression, among nurses worldwide (14, 15). Approximately 41.1% and 35.88% of nurses in China experienced anxiety and depression, respectively. (16, 17). However, the prevalence of anxiety and depression among neonatal department nurses and NICU nurses is still unknown. Furthermore, the effects of social factors and lifestyles on anxiety and depression among neonatal nurses remain unknown. Thus, this study aimed to explore the prevalence of anxiety and depression among neonatal nurses. Furthermore, this study analyzed the impact of social factors and lifestyle on mood disorders among neonatal nurses.

## Materials and Methods

### Study Design and Participants

This cross-sectional study was conducted using several questionnaires. The snowball sampling strategy was used to recruit the participants. The inclusion criteria were nurses who have no previous history of psychiatric diseases and who worked in the Department of Neonatology and the NICU of 12 general hospitals and 2 specialized hospitals affiliated to universities in Nanning city, Guangxi province, China. The questionnaire surveys were administered either by head nurses or neonatal nurses from January 1, 2022, to February 28, 2022. During this period, the participants could answer the anonymous questionnaire survey at any time; however, they could take the questionnaire survey only once and were required to answer all questions within 30 min. Written informed consent was provided by all participants before participating in the study; the study was approved by the Institutional Ethical Committee of Guangxi University of Chinese Medicine.

### Questionnaires

A validated Chinese version of a 7-item generalized anxiety disorder questionnaire (GAD-7) was administered to all the participants to identify the participants with generalized anxiety disorder, as defined by the DSM-5 (18). Researchers calculated each item score based on the following scale: a total score above five points can be identified as anxiety; namely, five–nine points indicated mild anxiety, 10–14 points indicated moderate anxiety, and scores higher than 15 points indicated severe anxiety.

The Chinese version of the Patient Health questionnaire-9 (PHQ-9) was used to screen the participants for depression, as defined by the DSM-5 (19). Researchers calculated each item score of the PHQ-9 based on the following scale: a total score above five points can be identified as depression. Specifically, five–nine points indicated mild depression, 10–14 points indicated moderate depression, 15–19 points indicated severe depression, whereas scores >20 points indicated severe anxiety.

The Chinese version of the Pittsburgh Sleep Quality Index (PSQI) was used to measure sleep quality. Sleep quality can be analyzed by measuring seven components over a period of 1 month, namely, subjective sleep quality, sleep latency, sleep duration, habitual sleep efficiency, sleep disturbances, use of sleeping medication, and daytime dysfunction. Researchers calculated each item score of the PSQI, as well as an overall sum of at least five indicators of poor sleep (20). Participants were excluded if they experienced any additional diseases according to the results of various tests, namely, electrocardiogram, abdominal ultrasound, X-ray, and blood tests from physical examinations conducted during annual check-ups. The endoscopy and diagnoses procedures were performed by a gastroenterologist.

The Rome IV diagnostic questionnaire for adult's functional gastrointestinal disorders identifies FD gastrointestinal symptoms among participants. Rome IV diagnostic questionnaire for measure postprandial distress syndrome (PDS) and epigastric pain syndrome (EPS), including postprandial fullness, early satiation, epigastric pain occurred in the last 3 months, and course of disease at least 6 months (21).

Social factors dates were self-reported by all participants as observed in their responses to the 11 items included in the questionnaires. These items comprise educational level (high school, or junior college, or undergraduate, or postgraduate), technical title (junior, or intermediate, or senior), years of working (1 year below, 1–5 years, 6–10 years, 10 years above), continuation of education (yes or no), head nurses (yes or no), interpersonal relationships (dissatisfaction, on average or satisfaction), previous work experience in the field of emerging infectious diseases (yes or no), marital status (single, married or divorced), spouse occupation (health care profession or non-healthcare profession), fertility status (nullipara, first births, second births or third births or above) and support from parents (yes or no).

Lifestyle dates were self-reported by all participants and the four items included in the questionnaire were preference for smoking (yes or no), alcoholism (yes or no), diet preference (coffee, tea, sugary beverages, chocolate, spicy, raw, deep-fried, hot, dairy products), and irregular diet (yes or no).

### Statistical Analysis

Statistical analyses were performed using the IBM SPSS Statistics version 25.0 to assess the study dates. Continuous variables such as age are presented as mean ± standard deviation. Categorical variables such as gender, diet preference and so on are expressed as proportions and percentages. The associations between relevant factors and study outcomes are presented as odds ratios (OR) and 95% confidence intervals (95% CI). Continuous variables were compared using Student's *t-test*. Categorical variables used *x*^2^ test, Fisher's exact test, or the *Ridit* test, as appropriate. Multivariable logistic regression analyses were used to identify the independent social and lifestyle factors associated with mood disorders. A two-sided *P*-value less than 0.05 was regarded as statistically significant.

## Results

### Prevalence of Mood Disorders Among Neonatal Nurses

A total of 260 nurses who had worked in the Department of Neonatology or the NICU of hospitals affiliated to universities participated in this study, including 8 (3.08%) males and 252 (96.92%) females, with ages varying between 20–48 years (30.0885 ± 5.43156).

Neonatal nurses' susceptibility to suffering from mood disorders, such as anxiety and depression has increased. In this study, 128 (49.23%) participants were affected by mood disorders, including anxiety or depression, with most nurses in neonatal suffering from mood disorders. Furthermore, 17 (13.28%) nurses had anxiety, whereas 29 (22.66%) nurses had depression, with 82 (64.06%) nurses exhibiting both anxiety and depression. Moreover, most participants with mood disorders exhibited mild anxiety and depression (Table 1).

**Table 1:** Prevalence of mood disorders among neonatal nurses (%).

**Degree**	**Anxiety**	**Depression**
Mild	74 (28.46)	77 (29.62)
Moderate	16 (6.15)	23 (8.85)
Moderate-severe	–	6 (2.31)
Severe	9 (5.45)	5 (1.92)
Total	99 (29.09)	111 (42.69)

### Effect of Social Factors on Neonatal Nurses With Mood Disorders

Various social factors have an impact on the mental health of neonatal nurses. This study confirmed that most of the nurses with mood disorders were female and had poor interpersonal relationships and an unhappy marital status. The prevalence was significantly higher than that of neonatal nurses without mood disorders (all *P* < 0.05) (Table 2).

**Table 2:** Demographic and social factors among neonatal nurses in mood disorders.

**Variables**	**Category**	**Nurses in mood disorders (%)**	**Nurses without mood disorders (%)**	
Gender	Male	1 (0.78)	7 (5.30)	
	Female	127 (99.22)	125 (94.70)	*x*^2^ = 105.679, *P* = 0.0000
Age		30.6484 ± 5.02997	29.5455 ± 5.76163	*t* = 1.646, *P* = 0.101
Educational level	High school	1 (0.78)	3 (2.27)	
	Junior college	20 (15.63)	20 (15.15)	
	Undergraduate	106 (82.81)	108 (81.82)	
	Postgraduate	1 (0.78)	1 (0.76)	*x*^2^ = 0.9574, *P* = 0.8116
Technical level	Junior	85 (66.41)	92 (69.70)	
	Intermediate	42 (32.81)	36 (27.27)	
	Senior	1 (0.78)	4 (3.03)	*x*^2^ = 2.4774, *P* = 0.2898
Years of working	1 year below	5 (3.91)	10 (7.56)	
	1–5 years	42 (32.81)	47 (35.61)	
	6–10 years	42 (32.81)	41 (31.06)	
	10 years above	39 (30.47)	34 (25.77)	*x*^2^ = 2.2411, *P* = 0.5239
Taking continuing education		47 (36.72)	54 (40.91)	*x*2 = 0.3201, *P* = 0.5715
Head nurse		3 (2.34)	6 (4.55)	*x*^2^ = 0.3989, *P* = 0.5276
Interpersonal relationship	Dissatisfaction	4 (3.13)	1 (0.76)	
	On average	56 (43.75)	40 (30.30)	
	Satisfaction	68 (53.12)	91 (68.94)	*x*^2^ = 7.7340, *P* = 0.0209
Previous work experience in anti-COVID-19		52 (40.63)	46 (34.85)	*x*^2^ = 0.6937, *P* = 0.4049
Marital status	Single	43 (33.59)	66 (50)	
	Married	82 (64.06)	66 (50)	
	Divorced	3 (2.34)	0 (0)	*x*^2^ = 5.2409, *P* = 0.0221
Spouse occupation	Health Care Profession	30 (35.29)	30 (45.45)	
	Non-health Care Profession	55 (64.71)	36 (54.56)	*x*^2^ = 1.2055, *P* = 0.2722
Fertility status	Nullipara	63 (49.21)	74 (56.06)	
	First births	36 (28.13)	30 (22.73)	
	Second births	28 (21.88)	27 (20.45)	*x*^2^ = 1.3856, *P* = 0.7089
	Third births or above	1 (0.78)	1 (0.76)	
Support parents		98 (76.56)	94 (71.21)	*x*^2^ = 0.9632, *P* = 0.3264

### Effect of Different Lifestyles on Neonatal Nurses With Mood Disorders

This study also confirmed that different lifestyles can affect the mental health of neonatal nurses. Specifically, smoking, alcohol consumption, and irregular diet were common among participants with mood disorders. Preference for both coffee and tea were lower among participants with mood disorders as opposed to the participants without (all P < 0.05) (Table 2).

**Figure 1:**
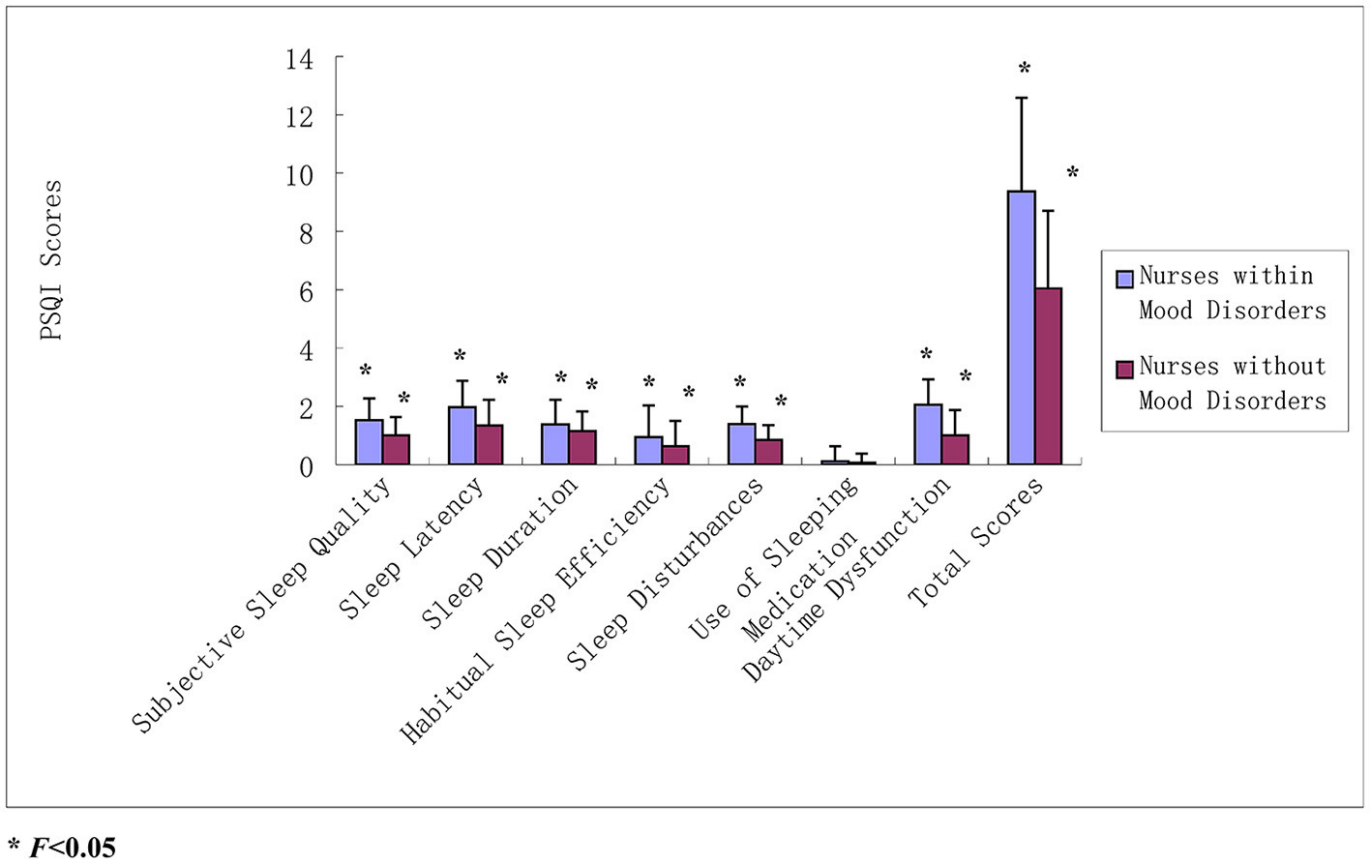
PSQI scores between neonatal nurses and mood disorders. All part of total PSQI scores and total PSQI scores among neonatal nurses with mood disorders were all higher than those without mood disorders. The differences in subjective sleep quality, sleep latency, sleep duration, habitual sleep efficiency, sleep disturbances, daytime dysfunction, and total PSQI scores (all *P* < 0.05).

Poor sleep has contributed toward mood disorders, as well as subjective sleep quality, sleep latency, sleep duration, habitual sleep efficiency, sleep disturbances, use of sleeping medication, daytime dysfunction, and total PSQI scores among neonatal nurses with mood disorders, which were all higher than those without mood disorders. The differences in subjective sleep quality, sleep latency, sleep duration, habitual sleep efficiency, sleep disturbances, daytime dysfunction, and total PSQI scores (all *P* < 0.05) were statistically significant (Table 3 and Figure 1).

**Table 3:** Lifestyles among neonatal nurses in mood disorders.

**Variables**	**Nurses in mood disorders (%)**	**Nurses without mood disorders (%)**	
Smoking	3 (2.34)	0 (0)	*x*^2^ = 123.0080, *P* = 0.0000
Alcohol	7 (5.47)	3 (2.27)	*x*^2^ = 110.3952, *P* = 0.0000
Diet preference			
Coffee	34 (26.56)	42 (31.82)	*x*^2^ = 19.1250, *P* = 0.0000
Tea	29 (21.97)	32 (24.24)	*x*^2^ = 33.2519, *P* = 0.0000
Sugary beverages	71 (55.47)	88 (66.67)	*x*^2^ = 3.4302, *P* = 0.0640
Chocolate	45 (55.47)	88 (66.67)	*x*^2^ = 0.0029, *P* = 0.9570
Irregular diet	85 (66.41)	62 (46.70)	*x*^2^ = 9.2155, *P*=0.0024
Poor sleep	113 (88.28)	75(56.82)	*x*^2^ = 30.5748, *P* = 0.0000

### Multivariable Logistic Regression Analysis on Independent Factors for Mood Disorders

Interpersonal relationships (OR = 0.530, 95%CI 0.326–0.860), marital status (OR = 1.849, 95%CI 1.763–1.940), irregular diet (OR = 1.972, 95%CI 1.880–2.069) and poor sleep (OR = 7.295, 95%CI 6.850–7.770) were selected as independent factors associated with mood disorders among neonatal nurses (all *P* < 0.05) (Table 4). These results suggest that good interpersonal relationships are an independent protective factor for mood disorders in neonatal nurses. Unhappy marriage or divorce, irregular diet, and poor sleep are independent risk factors for mood disorders in neonatal nurses.

**Table 4:** Multivariate logistic regression analyses on selected factors associated with mood disorders.

**Selected factors**	**Mood disorders**		
	**OR**	**95%CI**	***P-*value**
Gender	3.936	(3.337, 4.643)	0.373
Interpersonal relationship	0.530	(0.326, 0.860)	0.044
Marital status	1.849	(1.763, 1.940)	0.009
Alcohol	2.028	(1.770, 2.324)	0.375
Coffee	0.757	(0.719, 0.798)	0.253
Tea	1.054	(0.995, 1.116)	0.838
Irregular diet	1.972	(1.880, 2.069)	0.025
Poor sleep	7.295	(6.850, 7.770)	0.000

### Prevalence of FD-Associated Mood Disorders Among Neonatal Nurses

Among 128 nurses with mood disorders, 65 (50.78%) presented FD symptoms, which was higher than those without mood disorders (50.78% vs. 11.48%; *P* < 0.05). Most of them (53.85%) had PDS, but there was no difference (*x*^2^ = 3.2347, *P* = 0.1984) (Table 5).

**Table 5:** Prevalence of FD in mood disorders among neonatal nurses (%).

**Variables**	**Nurses in mood disorders**	**Nurses without mood disorders**
FD^*^	65 (50.78)	14 (11.48)
EPS^#^	17 (26.15)	1 (7.14)
PDS^#^	35 (53.85)	11 (78.57)
Both EPS and PDS^#^	13 (20)	2 (14.29)

* *Prevalence of FD between nurses within and without mood disorders: x^2^ = 42.8479, P = 0.000*;

# *Prevalence of FD subtypes between nurses within and without mood disorders: x^2^ = 3.2347, P = 0.1984*.

### Lifestyle Impact on FD-Associated Mood Disorders

This study showed that different lifestyles exhibit varying impacts on mood disorders associated with FD. The prevalence of smoking and poor sleep was significantly higher in patients with FD and mood disorders than in those without mood disorders (all *P* < 0.05). However, the prevalence of preference for sugary beverages was lower in participants with mood disorders associated with FD (*P* < 0.05) (Table 6).

**Table 6:** Lifestyles among neonatal nurses in FD with mood disorders.

**Variables**	**FD within mood disorders (%)**	**FD without mood disorders (%)**	
Smoking	1 (1.54)	0 (0)	*x*^2^ = 0.7237, *P* = 0.03949
Alcohol	2 (3.08)	0 (0)	*x*^2^ = 0.0746, *P* = 0.7848
Diet preference			
Coffee	14 (21.54)	5 (35.71)	*x*^2^ = 0.6100, *P* = 0.4348
Tea	15 (23.08)	5 (35.71)	*x*^2^ = 0.4194, *P* = 0.5173
Sugary beverages	14 (21.54)	10 (71.43)	*x*^2^ = 11.2995, *P* = 0.0008
Chocolate	21 (32.31)	7 (50)	*x*^2^ = 0.8974, *P* = 0.3435
Spicy food	38 (58.46)	6 (42.86)	*x*^2^ = 0.5923, *P* = 0.4415
Row food	17 (26.15)	2 (14.29)	*x*^2^ = 0.3573, *P* = 0.5500
Deep-fried food	18 (27.69)	4 (28.57)	*x*^2^ = 0.80687, *P* = 0.7933
Hot food	15 (23.08)	3 (21.43)	*x*^2^ = 0.0475, *P* = 0.8275
Dairy products	18 (27.69)	3 (21.43)	*x*^2^ = 0.80218, *P* = 0.8825
Irregular diet	51 (78.46)	8 (57.14)	*x*^2^ = 1.7561, *P* = 0.1851
Poor sleep	61 (93.85)	10 (71.43)	*x*^2^ = 4.1359, *P* = 0.0420

**Figure 2:**
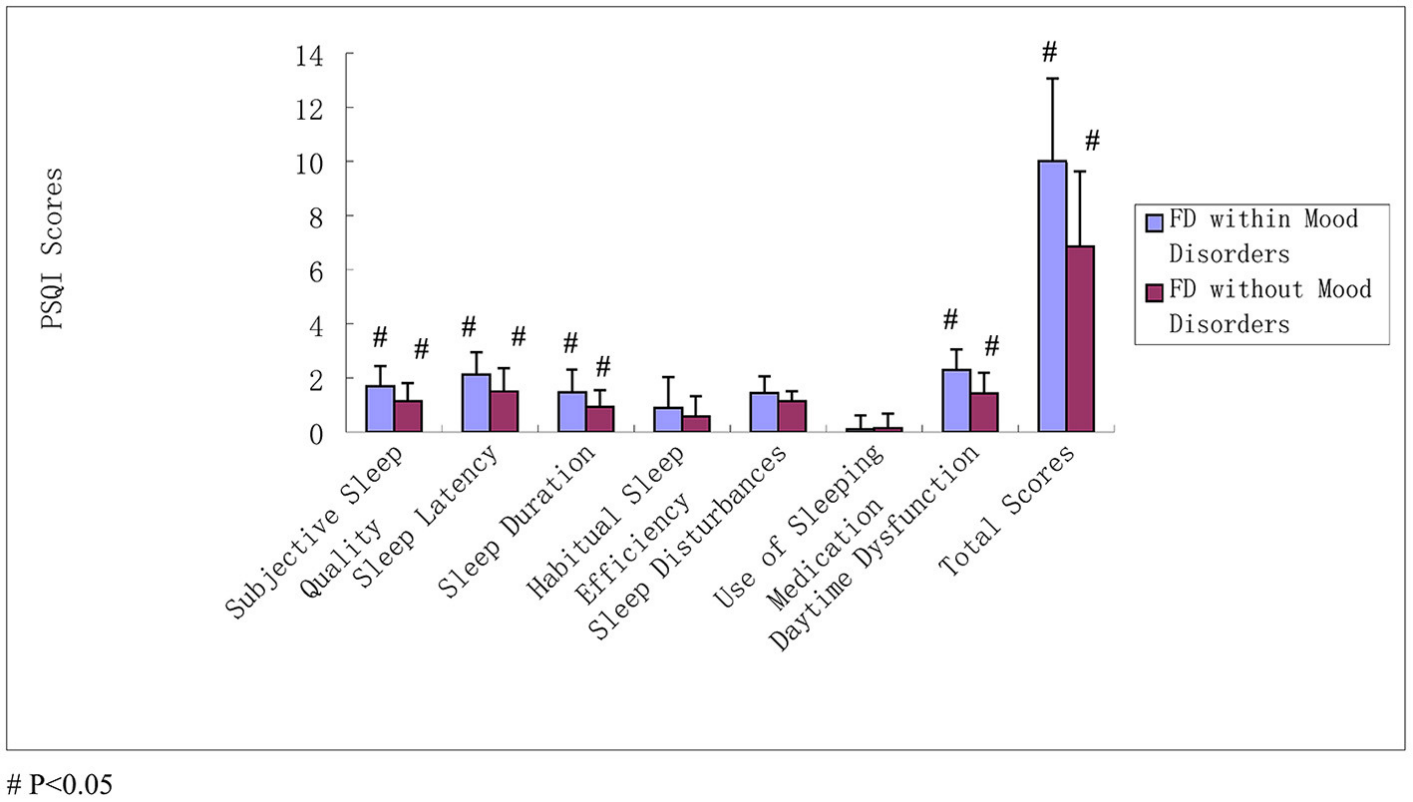
PSQI scores between neonatal nurses in FD and mood disorders. The scores of subjective sleep quality, sleep latency, sleep duration, habitual sleep efficiency, sleep disturbances, daytime dysfunction, and total PSQI scores among neonatal nurses with FD-associated mood disorders were all higher than those exhibiting FD without mood disorders; the subjective sleep quality, sleep latency, sleep duration, daytime dysfunction, and total PSQI scores were statistically significant difference (all *P* < 0.05).

Poor sleep also displayed effects on FD-associated mood disorders. The scores of subjective sleep quality, sleep latency, sleep duration, habitual sleep efficiency, sleep disturbances, daytime dysfunction, and total PSQI scores among neonatal nurses with FD-associated mood disorders were all higher than those exhibiting FD without mood disorders. There was a statistically significant difference among the subjective sleep quality, sleep latency, sleep duration, daytime dysfunction, and total PSQI scores (all *P* < 0.05) (Table 6 and Figure 2).

### Multivariable Logistic Regression Analysis on Independent Factors for Mood Disorders With FD

A multivariable logistic regression model was performed to identify the association between lifestyle factors and FD-associated mood disorders. Consequently, poor sleep (OR = 6.10, 95%CI 1.309–28.419, *P* < 0.05) was identified as an independent risk factor associated with FD among neonatal nurses (Table 7).

**Table 7:** Multivariate logistic regression analyses on selected factors associated with FD and mood disorders.

**Selected factors**	**Mood disorders**		
	**OR**	**95%CI**	***P-*value**
Sugary beverages	0.503	(0.160, 1.985)	0.179
Poor sleep	6.10	(1.309, 28.419)	0.013

## Discussion

Nurses are one of the frontline health workers for patient care, especially newborn infants. Newborn infants have decreased habituation and dishabituation capabilities, resulting in increased environmental vulnerability. Thus, patient care is more critical among newborn infants (22). However, neonatal nurses are often overworked and stressed by the heavy workload pressures associated with clinical care. As a result, nurses are more likely to experience mood disorders such as anxiety and depression (1). Furthermore, several studies have confirmed a high prevalence of mood disorders such as anxiety and depression among nurses; in China, approximately 35% of the nurses experienced depression, while 32–43% have anxiety (23–25). In a recent study, 49.23% of neonatal nurses experienced mood disorders, including anxiety and depression. From this sample, 31.54% experienced both anxiety and depression, which was relatively higher than other departmental nurses as well as the general population (26).

Previous studies confirm that social and lifestyle factors can impact mood disorders, such as anxiety and depression (24–27). This study found that poor interpersonal relationships, unhappy marital status, high levels of smoking and alcohol consumption, irregular diet, and poor sleep increased the susceptibility of experiencing anxiety and depression among neonatal nurses. In contrast, coffee and tea were identified as having a protective effect against mood disorders. Following the multivariable logistic regression analysis, good interpersonal relationships were identified as an independent protective factor associated with mood disorders in neonatal nurses. Whereas unhappy marital status, irregular diet, and poor sleep were all identified as independent risk factors associated with mood disorders in neonatal nurses.

Individuals with depression tended to interact with others in ways that elicit rejection, typically characterized by poor interpersonal relationships; furthermore, these nurses have been associated with an increased occurrence of negative interpersonal dependent events, which, in turn, increase the risk of future depression and anxiety (28, 29). Moreover, anxiety and depression were also independently associated with irregular diet and sleep (30–33). Sleep disorders, including subjective sleep quality, sleep latency, sleep duration, habitual sleep efficiency, sleep disturbances, use of sleeping medication, and daytime dysfunction, have all been found to affect mood disorders (34). Night-shift work is common among neonatal nurses which often affects nurses' sleep rhythm and sleep quality. As a result, neonatal nurses experience poor sleep which may have an impact on anxiety and depression.

Recent findings have also confirmed that smoking is regarded as a false safety behavior that has led to anxiety and depression. Consequently, mood disorders further exacerbate smoking behavior (35, 36). Furthermore, alcohol can influence the severity of moods experienced. While negative mood symptoms can disappear in a short period, mood disorders can result in alcohol misuse. Moreover, family factors, poor family interpersonal relationships, or unhappy marital status can all impact alcohol misuse and the occurrence of mood disorders (37–39). In contrast, coffee and tea have potential protective effects against depression. Caffeine, chlorogenic acid, and 5-hydroxytryptamides in coffee and tea can increase calcium signaling and dopamine release, thus, forming protection against mood disorders (40–42).

Gastrointestinal sensory mechanisms play a key role in transferring sensory information from enteric reflex circuits to the central nervous system (CNS) via the vagal and spinal nervous systems. Furthermore, CNS has a significant effect on the gastrointestinal tract. Functional gastrointestinal disorder is a psychosomatic disorder. FD is one of the most common physical symptoms of psychosomatic disorders (43–45) and is divided into two subtypes according to abdominal symptoms: postprandial distress syndrome (PDS) and epigastric pain syndrome (EPS).

In this study, 50.78% of neonatal nurses experienced anxiety and depression presented through FD. Most of them had PDS (53.85%), followed by EPS (26.51%). Whereas a few of these nurses had both PDS and EPS (20%). There was a statistically significant difference between the prevalence of FD with and without mood disorders. However, no difference was observed between the two subtypes of FD, with and without mood disorders. Another study confirmed that the prevalence of anxiety and depression in patients with FD was higher than in the healthy population (46). Psychiatric comorbidity was common among the patients who presented with FD referred to the Department of Gastroenterology. Further, psychiatric comorbidity increased the number, frequency, and severity of gastrointestinal symptoms associated with functional gastrointestinal disorders (FGIDs) (47).

Lifestyle factors also play an important role in FD-associated mood disorders among neonatal nurses. This study found that neonatal nurses who preferred smoking and poor sleep were more likely to suffer from FD associated with anxiety and depression. Based on the multivariable logistic regression analysis, poor sleep was identified as an independent risk factor associated with FD and mood disorders among neonatal nurses. Furthermore, other studies have suggested that sugar-sweetened beverage consumption is one of the modestly high-risk factors underlying depression. The current study did not note any differences; thus, further studies are required to confirm otherwise (48).

This study had several limitations. First, this was a cross-sectional study to explore the effects of social and lifestyle factors on mood disorders and FD solely among neonatal nurses, thus, limited information was available. Second, the self-report questionnaire used in this study may have caused some deviation in the results or the information could be false. Finally, most of participants were female in this study, it may cause deviation in the result of gender factor.

To conclude, this study confirmed that the prevalence of anxiety and depression among neonatal nurses is significantly high, as opposed to other departments' nurses and the public. Furthermore, most of the psychiatric comorbidities in participants commonly presented as FD. Thus, social and lifestyle factors play a key role in mood disorders among neonatal nurses. Moreover, social and lifestyle factors have a significant impact on FD, anxiety, and depression in neonatal nurses.

## Data Availability Statement

The original contributions presented in the study are included in the article/supplementary material, further inquiries can be directed to the corresponding author/s.

## Ethics Statement

The studies involving human participants were reviewed and approved by the Institutional Ethical Committee of Guangxi University of Chinese Medicine. The patients/participants provided their written informed consent to participate in this study.

## Author Contributions

Z-pH and FH designed the study. Z-pH, FH, M-jW, C-zT, J-pH, JL, S-eL, S-qW, H-hL, J-jL, and XL performed the experiments. Z-pH acquired and analyzed the data and wrote the manuscript. All authors contributed to the article and approved the submitted version.

## Conflict of Interest

The authors declare that the research was conducted in the absence of any commercial or financial relationships that could be construed as a potential conflict of interest.

## Publisher's Note

All claims expressed in this article are solely those of the authors and do not necessarily represent those of their affiliated organizations, or those of the publisher, the editors and the reviewers. Any product that may be evaluated in this article, or claim that may be made by its manufacturer, is not guaranteed or endorsed by the publisher.
